# Ethics, virtues and xenotransplantation

**DOI:** 10.1177/02676591221140767

**Published:** 2022-11-16

**Authors:** Andrew JT George

**Affiliations:** Department of Surgery and Cancer, 170714 Imperial College London, London, UK

**Keywords:** xenotransplantation, ethics, virtue ethics, telos, deontology, consequentialism

## Abstract

Early in 2022 the first pig to human cardiac xenotransplant was performed. The graft initially performed well, and rejection was well controlled. However, the graft failed, and the patient died 60 days after the procedure. The ethical issues relating to xenotransplantation include the risk/benefit to the individual, the risk of porcine-derived infectious agents crossing into humans, animal welfare and rights, issues of human and animal identity and concerns relating to fair allocation of organs and appropriate use of resources.

These ethical issues are often addressed using emotional arguments, or through consequentialist or deontological lens. An alternative is to use approaches based on virtue ethics to understand the moral purpose (*telos*) of the research and the virtues (character traits) needed to be a good research clinician. In this review we will consider the virtues of justice, courage, temperance and practical wisdom, as well as the role of clinical curiosity, and their application to xenotransplantation. This provides an alternative approach for the clinical academic and others involved in the research to reflect on their practice.

## Introduction

One of the remarkable advances in treating end stage organ failure has been the development, starting in the second half of the 20th century, of transplantation.^1^ The strength of the alloimmune response meant that initially success was only seen when vascularised transplants were performed between genetically identical donors and recipients^2,3^ (it should be noted that transplantation has been successfully performed for non-vascular organs such as the cornea since 1906).^4^ However, the advent of HLA matching, together with the development of immunosuppressive drugs, has meant that transplantation has become the treatment option for many forms of end stage organ failure.^5^

The major limitation of conventional allotransplantation is the shortage of organs. While it is important to increase the donor pool (for example by increasing use of living donors and increasing potential donor registration), it is unlikely that there will ever be enough organs to meet the needs of all potential recipients.^6^ For example, median survival of patients who have received a cardiac transplant is greater than 12 years^7^ and transplantation is therefore the gold standard for many patients with refractory heart failure.^8^ This suggests that if there was an unlimited supply of organs that the indications for organ transplantation would increase.

One source of organs could be other species. The potential for xenotransplantation to supply the needs for organs has excited considerable research. Much of the initial research focussed on using non-human primates as donors.^9,10^ However, while this approach appears logical given the genetic similarity between donor and recipient, the primate is not an ideal donor. In large part this is because they are in relatively short supply, there is an increased risk of zoonotic infections and many have moral concerns about the use of animals that are close to us in evolutionary terms as transplant donors.^11^

This has led to a focus on the pig as a potential donor. This species is widely farmed for food (estimated 784 million pigs currently farmed worldwide).^12^ They grow and reproduce rapidly. There is a considerable (though not total) degree of anatomical and physiological compatibility between pigs and humans.^13^

The development of approaches that allow the cloning and genetic manipulation of the pig,^14^ has allowed the development of new approaches to preventing or mitigating immune rejection.^15,16^ These approaches might also allow the induction of tolerance to the xenograft, thus reducing the need for long term immunosuppression and the consequent side effects.^16,17^

The nature of the immune response against a xenograft is multifactorial.^11^ The first mechanism that leads to graft hyperacute rejection is the existence in humans of pre-circulating natural antibody against the pig graft. These, which largely recognise the carbohydrate galactose α-1,3 galactose,^18,19^ bind to sugar moieties on endothelial cells and lead to graft rejection in minutes. While the galactose α-1,3 galactose epitope is dominant, others have also been identified. There are also molecular incompatibilities, for example in molecules that control coagulation that increase the thrombotic risk.^20^ In addition, acute T cell mediated rejection and chronic rejection (both cellular and antibody mediated) would lead to graft rejection if not controlled.

The history of xenotransplantation has been well summarised in a number of publications.^9,10^ The progress to clinical application has been somewhat slower than many (including this author) imagined. However, there has been considerable progress in recent times.^16^ This has been developed using a number of preclinical porcine to non-human primate models.^21–24^ In two studies porcine kidneys (in one case following removal of the gene encoding α 1.3-galactosyl transferase, responsible for the galactose α-1,3 galactose epitope and in the other 10 genetic modifications as described below for clinical cardiac xenotransplantation) have been grafted into brain dead human recipients with no signs of hyperacute rejection during the (inevitably) short (up to 72 hours) duration of the experiment.^25,26^

The first gene modified pig to human cardiac transplant was carried out in January 2022.^27^ The patient had severe heart failure (left ventricular ejection fraction of 10%) and was being treated with extracorporeal membrane oxygenation. He was not a candidate for allotransplantation, denied by 2 regional and 2 national transplant programmes due to poor adherence to treatment. The donor pig carried 10 modified genes. Three of these involved deletion of genes (termed gene knockout) in order to remove natural xenogeneic carbohydrate antigens (α-1,3-galactosyl transferase responsible for the galactose α-1,3 galactose epitope, β-1,4-N-acetyl-galactosyl transferase responsible for the SDa blood group and CMP-*N*-acetylneuraminic acid hydroxylase responsible for N-glycolylneuraminic acid). One gene knockout (growth hormone receptor) was introduced to reduce growth of the graft. Two human genes (CD46 and Decay Accelerating Factor) were introduced to downregulate complement activation, two genes (Endothelial Cell Protein C Receptor and Thrombomodulin) for anti-coagulation and two genes (haem oxygenase 1 and CD47) to reduce inflammation. The animal was negative for porcine endogenous retrovirus C (PERV-C) and was screened for other relevant porcine viruses. Immunosuppression included agents to deplete T and B cells (Rituximab and anti-thymocyte globulin), inhibit complement activation and block CD40 co-stimulation.

The patient was supported by the porcine heart for 7 weeks before a sudden unexplained deterioration in function which resulted in withdrawal of life support on day 60.^27^ Until the loss in function there had been no obvious immune rejection of the graft. The patient did show unexpected evidence of porcine Cytomegalovirus. While this attempt did not result in long term survival, this experimental approach will produce data that will inform future work in making xenotransplantation of practical, clinical utility.^28^ It is perhaps worth comparing this experience to the first cardiac allotransplants in the mid-1960s that typically lasted a few weeks.^29^

## The ethics of xenotransplantation

Xenotransplantation has raised considerable ethical discussion and has led to the production of guidelines and regulatory documents by a number of authoritative professional and governmental organisations.^30^ In general, the ethical debate has been informed by three ethical approaches; emotional, deontological and consequentialism. To a large extent the main lines of the ethical debate have been fixed for several decades. In this paper I will review some of the current debate, before looking at a different ethical framework, that of virtue ethics, to examine how that might inform the practice of scientists and doctors in the field of xenotransplantation.

Some of the major ethical issues in xenotransplantation are shown in Table 1. These include the risk of porcine-derived infectious agents causing disease in humans;^31^ awareness of the risk of zoonotic disease has been heightened by the COVID-19 pandemic.^32^ Other issues relate to the impact on the patient, the animal donors, identity and also issues relating to the allocation of organs and whether this is a resource effective solution to health challenges. In addition to these, ethical issues common to all clinical practice and research naturally apply equally to xenotransplantation.^33,34^

**Table 1.** Potential ethical issues in xenotransplantation. Selected recent references are given to inform further reading.

**Risk benefit for patient**
Physical: Is the procedure likely to prolong good quality life?^13,30^
Psychological: Will the patient suffer identity issues?^35^
**Burden to patient**
Lifelong monitoring: What harm due to need to monitor patient?^30,36–38^
**Zoonotic disease**
Risk to population: Risk of developing novel zoonotic infectious agent.^30,36–38^
**Animal welfare**
Breeding donor animals: Will the animals be maintained in a humane way?^37–40^
**Animal rights**
Exploitation of animals: Is it right to modify animals for treating humans?^38,40^
**Identity**
Human-animal chimeras: Is there a threat to human or animal identity?^39,41,42^
**Justice**
Access to organs: Will organs be available to all groups in society?^13,40,43^
Use of resource: Is there a better approach to health?^13,37^

## Emotional

Much of the response to xenotransplantation is an emotional response. People are either drawn to the concept or repulsed by the very idea of putting animal tissue in humans. These emotional responses have been of long standing. Many cultures have been fascinated in chimeras, fantastical mixtures of different animals.^44^ In equal measure, people have been long been horrified when medicine has been seen to cross certain boundaries; much of the antagonism to Jenner’s cowpox vaccination stemmed from a belief it went against God and nature.^45^

Xenotransplantation excites an emotional response in many ways.^35,46^ Some people see interfering with the genetic makeup of pigs, especially if that involves the introduction of human genes, as crossing some form of moral boundary. For some this will be because scientists are disturbing the intrinsic nature of the species. Advocates of xenotransplantation can point to the fact that humans have been changing the genetic nature of other animals by selective breeding ever since the first animals were domesticated. This selective breeding has had a far greater effect on porcine genes than the relatively small number of alterations introduced to frustrate the host inflammatory response.^47^

However, emotions can run in both directions, and emotional appeals are also employed by scientists and doctors.^39,46^ Proponents of xenotransplantation are often motivated by the acute need of patients which can propel them into reaching for a simple solution to what is actually a complex problem (the patient dying of end stage organ failure has, in most cases, been on a long disease pathway; interventions earlier in that pathway might be more effective,^48^ but do not command the same emotional weight as performing a transplant).

While emotional responses might seem to lack an intellectual rigour, they are very influential. Indeed, we make many of our ethical decisions on an instinctive, emotional, basis and then apply *post hoc* reasoning to justify our decision. One influential school of ethical reasoning, termed emotivism, argues that ethical statements are in fact statements of preference and not statements of fact. Declarations such as ‘I approve/do not approve of xenotransplantation’ describe the emotional attitude of the speaker and are not amenable to proof or argumentation.^49,50^ While this argument has largely fallen out of favour in academic circles, the emotional component is important in much day-to-day ethical decision making.

## Consequentialist

The major ethical frameworks used in medical and scientific contexts are consequentialist and deontological.^50^ Consequentialism uses an approach of considering the benefits and dis-benefits of any action and then attempting to weight them up. One should aim to maximise the benefits and minimise the harms of any action.

This approach is often used in research. In the context of xenotransplantation consideration of the benefit to any patient (due to receiving a functioning organ) and the harm that they might have (the interventions and therapy relating to the approach as well as the harm of the transplant failing) have to be carefully considered.

One issue that needs to be carefully considered is how to measure the relative benefits or harms. In the case of the individual patient that can be theoretically relatively simple (there is a risk of death for a patient that does not receive a xenograft that can be compared to the risk of death if they did). However, in xenotransplantation these comparisons are complicated by the unknown nature of outcomes inherent in research. Some of the comparisons are more difficult because one is comparing different parameters that will be valued by people differently (how does one compare the harm caused by the side effects of therapy to the psychological benefit that hope brings to the patient?). The benefits and harms may fall on different people (the benefit of an individual having a longer life is compared to the risk of a zoonotic infection for third parties). These issues are to some extent addressed by different forms of consequentialism that understand the benefits and harms in different ways.^50^

Philosophers have also pointed out that the pure application of consequentialist arguments can produce perverse results.^51^ One example is to assume that a rape has been carried out in a neighbourhood. The populace is angry and beginning to riot, causing loss of life. Your testimony could result in the arrest of a suspect. You know that they are innocent, but your false testimony would bring an end to the riots and save lives. It is possible to use a consequentialist argument to support framing this innocent suspect because the benefit that will be achieved by stopping the rioting and consequent loss of life outweighs the harm to that individual by deprivation of their liberty. Most people would consider this action would not be ethical and that the consequentialist argument has produced a perverse outcome.^50^

There is the potential for similar dilemmas in transplantation. As will be discussed below the survival of recipients of the first cardiac allografts was very poor,^29^ and it is questionable whether the procedure was in their best interests. Similarly early renal allografts showed poor graft survival.^1^ However, these early studies paved the way for modern transplantation. A consequentialist might hold that this harm was justified by the benefit that the procedure has bought to so many people. It is dangerous to use such arguments in isolation!

## Deontological

Deontology effectively derives rules, duties or guidelines for conduct. These guidelines can be derived from authority (historically often a religion) or from imperatives such as those developed by Kant using reason (for example; ‘Act in such a way that you always treat humanity … never simply as a means but always at the same time as an end.’^52^)

These guidelines can take the form of a list of duties for doctors, or a series of rules that need to be followed in certain circumstances. These are often derived from professional bodies, and a number of guidelines have been developed for xenotransplantation by relevant authorities, as reviewed in.^30^

There are several issues with a deontological approach. One is the authority of the body issuing the rules or guidelines. In the absence of a generally accepted religious framework, it is problematic to locate that authority in a divine being. In general, the authority of the professional bodies springs from either a democratic mandate (deriving from an elected government) or by the process that has been used to generate the guidelines (demonstrating the right expertise, public engagement and level of consultation in their production). However, they are always susceptible to the ‘Says who?’ challenge.

A further issue is that these guidelines encourage a ‘good enough’ approach to a subject. In general, if you follow guidelines, you will behave safely and competently. But they do not encourage excellence of conduct.

Guidelines only address issues that are known. Many ethical dilemmas occur in grey zones where either guidelines have not been written or where there is ‘guideline conflict’, with the rules and duties being in conflict. One solution to this issue is to produce more guidelines to resolve or cover these areas. This can lead to a mushrooming of rules and regulations that creates confusion and consumes resource.

It is also worth considering that the use of lists and guidelines can encourage practitioners to outsource their ethical reasoning. All they have to do to be ethical is to follow the rules that others have written, rather than think for themselves.

## Principles

A solution to some of these issues is to develop principles. These can be used by individuals and organisations to guide them as to what to do. In clinical medicine the four principles that are commonly used are; respect for autonomy, non-maleficence, justice and beneficence.^53^

While there can be ‘principle conflict’, principles are a helpful approach to giving doctors and scientists a framework to think through their actions. For example, when considering xenotransplantation the principle of respect for autonomy argues for informed consent regarding the health risks, non-maleficence includes public health risks, justice the fair allocation and animal rights while beneficence is concerned with the reduction of harm and the maximisation of benefits.^41^

## Virtue ethics

A further approach to ethical reasoning is the use of virtue ethics. This is an ancient form of ethical thinking, pioneered by Aristotle,^54^ that was the major ethical framework used in the Western World until the advent of consequentialist and deontological approaches in the Enlightenment.^49^ It differs from the other approaches in concentrating on the character of the person, rather than their actions. At its (over) simplest it can be characterised as arguing that good people do good things.

### Telos

There are several important components to virtue ethics. One is the moral purpose or ultimate end (*telos*) of the person. This is what the individual should strive for. There should be no difference between the moral and practical purposes of an individual – they should be the same.^55^ A good action is one that is both morally good and also successful. The *telos* of a doctor could be described as ‘helping people flourish through improving health’.^56^ A successful doctor is therefore someone who achieves this aim.

In the context of experimental xenotransplantation (and indeed all forms of clinical academia) there needs to be further consideration of the *telos* of the scientists and surgeons/doctors involved. A ‘pure’ (non-clinical) scientist may not see their *telos* as ‘helping people flourish through improving health’, but rather ‘helping society flourish by improving knowledge or understanding’. How does that align with the *telos* of the clinician?

One answer might be to assume that a clinical academic has two ultimate ends that they are striving towards, one clinical and the other scientific. This is unsatisfactory, having multiple purposes leads to tensions and a danger that one fails to achieve all or any of them. Having two ‘ultimate’ ends is also, on the face of it, a contradiction.

An alternative is to examine the *telos* of the clinician; ‘helping people flourish through improving health’. In this context who are ‘people’? For most clinicians it will be the patient in front of them, their family and, possibly, the population that they serve. For clinical academics this may include people with a particular condition or disease, or people who will be patients in the future, rather than present.

The clinical academic may also have a wider understanding of how improving health can result in flourishing. For most clinicians this will involve some form of diagnosis and intervention or treatment of the individual or population. For the clinical academic this can also involve improving understanding of pharmacological, pathological, physiological, psychological and sociological mechanisms and also developing and evaluating interventions seeking to improve health.

While a clinical academic may interpret their *telos* in this way, their *telos* must also embrace that of the patient in front of them. A virtuous clinical academic would not (for example) perform a xenotransplant that they know will harm their patient in order to produce knowledge that will benefit future patients.

In much biomedical research there will be many members of the research team (the paper describing cardiac xenotransplantation had 13 authors and more than 50 personally named as contributing to the work as well as unnamed members of various teams^27^). They will have their own objectives. Some will be personal (career progression, job satisfaction, sense of achievement). In addition, their professional purposes may be different. It is to be expected that the surgeons and other clinicians would have the development of improved treatment and care for their patient at the heart of what they do. Scientists in the team may be more driven by the desire to increase knowledge and understanding. Business representatives will be appropriately motivated by commercial success. These can all be good and honourable purposes, but in clinical research the final (ultimate) moral purpose of the project must relate to improving health, so enabling human flourishing. This should be a shared *telos* for all involved in clinical research.

Consideration of their *telos* in the context of the recent report of cardiac xenotransplantation,^27^ should prompt the clinical academic to consider several issues. They would need to consider if the transplant is likely (given the state of knowledge at the time) to benefit the health of the recipient, whether the risk/benefit ratio for the individual is favourable. They would also need to consider if the research would help future patients (whether it is well designed). In the case of xenotransplantation, they must consider the risks to the health of others, most notably through the development of novel infectious agents. They might also consider if there are alternative approaches that would be better (e.g., mechanical devices), and possibly (particularly when deciding what research to do) whether there is a better route to health and flourishing (such as preventative approaches).

## Virtues

The virtues of the doctor are those character traits that contribute to them reaching their *telos*. In classical virtue ethics there were four cardinal virtues (though many others have been described by writers, including Aristotle^54,55^). These are temperance, justice, courage and practical wisdom.^57^ A virtuous person has developed these traits and orchestrates them appropriately using their practical wisdom.

### Temperance

This virtue describes the character of self-control or self-restraint. It is a vital virtue for clinicians, including researchers. Just because one can do something, it is not always the best thing to do it. A virtuous clinician is one who knows when not to do something!

In the context of xenotransplantation, temperance would be necessary for the researcher to gauge the relative benefits of carrying out a procedure. There is a lot of pressure on doctors to develop their self-esteem, their reputation, to demonstrate their prowess, to ‘do something’ for their patient, to be the first, to publish and to get grant money.

This virtue, like all virtues, is about maintaining an appropriate balance. A doctor who shows too much temperance may hold back from doing something that they should do. They should know when to engage this virtue and when not to. For example, the motivation for a researcher to be the first to carry out a particular procedure is not a bad motivation; such motivations drive progress. However, when considering whether to perform that procedure a researcher should show a high degree of temperance; the risk to the patient demands that they show considerable caution and self-restraint. When discussing their research findings (for example at a conference) it is necessary to use a different form of temperance which, while promoting the researcher’s argument, allows them to listen and learn from others.

The virtue of temperance is often informed by interactions with others involved in the process. A clinical researcher who is exhibiting temperance will be open to hearing the voices of their colleagues (academic and care), other researchers and patients and using the resulting network of differing thoughts to inform and moderate their actions.

In the example of cardiac xenotransplantation,^27^ temperance would have led to the research team carefully considering the potential risks and benefits to the patient and others and not be overly motivated by a desire to ‘be the first’. They would have been open to the research findings of other groups, considering whether there might be a better approach. Temperance may also have contributed to the decision that further treatment would be futile, ceasing life support rather than continuing heroic treatment.

### Justice

There are two aspects of justice: distributive justice (ensuring that different people are treated fairly) and giving people what is due to them (making sure that the needs and wants of patients are considered). For doctors and researchers, the virtue of justice will embrace the patients, the wider population, the teams around the patient, themselves and also other research groups.

The needs and wants of the patient will embrace things such as the information that they are given as part of the experimental treatment. The doctor may need to also find out what the health needs of their patient is; what do they need to flourish. A just researcher will seek to make sure that recruitment to studies is equitable. They will seek to ensure that other members of their research and clinical team, and other research groups, get what is due to them in terms of recognition and support.

Justice extends to their own interests, they should render to themselves what is due to them, which may involve cultivating their career, job satisfaction and personal development. Justice needs to be held in balance. While the researcher should be concerned about what is due to them (and others) this needs to be in moderation and balanced appropriately.

The recipient of the cardiac xenograft was due several things. One would be that their needs were fully considered and the research team took account of what their long term requirements and desires (what would health and flourishing mean for this individual, who had history of non-compliance, and how might that be met by the transplant?). They should also receive the information to enable them to understand the potential benefits and consequences of the therapy (including long term medication and follow-up).

Justice would also motivate the researchers to question whether the introduction of xenotransplantation might be unfair to certain groups in society.^40^ This would include the question as to whether this is a treatment option that would be unaffordable by some, or whether it would divert resource from other treatments. There is also a possibility that in future pigs will be genetically modified to so as to increase compatibility with certain groups of people (for example, those that share a particular tissue or HLA type). This could discriminate against certain groups.

### Courage

Courage is a virtue that is widely seen as essential in all walks of life. The way in which courage manifests itself is different; the courage of a surgeon performing an operation for the first time is different from that of a soldier on the front line.

Courage is clearly needed to do something that has risk in it. A good doctor will understand their courage and moderate it appropriately. If they are a coward, they will not be a good doctor, but if they are foolhardy, they will take unnecessary risks and so also not be a good doctor. As described for the other virtues, the extent and nature of courage needs modulation according to circumstances; removal of the patient’s existing heart must require courage, while treating post-operative sepsis requires a greater degree of caution.

Courage would have been needed at several stages in developing cardiac xenotransplantation. Courage would have been necessary to initiate such a long-term and ambitious project. It would take extreme courage to carry out the surgery for the first time. It would have taken courage to have frank conversations with the recipient and their family as the graft deteriorated and finally the decision was made to cease life support. These three instances of courage are both quantitatively and qualitatively different from each other, but are all needed to reach the purpose of good research.

### Practical wisdom

The researcher must know how to bring in their different virtues appropriately, to achieve their *telos*. They also need to understand how and when to use their technical skills. To do this they require practical wisdom (in ancient Greek, *phronesis*), which they can use to orchestrate their skills and virtues in the best way.^55^

The practical wisdom is developed by experience, curiosity, reflection and by observation (of role models or leaders). It is perhaps the most important of the virtues. Certainly, without technical knowledge it is not possible to be a ‘good doctor’. It will be the wisdom of the researcher that will enable them to gauge if the state of knowledge, the patient’s condition and other factors mean that the time is right to try the novel procedure.

Practical wisdom in experimental xenotransplantation would orchestrate the virtues and skills appropriately. It is not that all the virtues need to be in balance at all times. For example, the precise moment of surgically removing the recipient heart and replacing it with the porcine donor would require all the surgeon’s technical skill and courage. There is probably little need, at that time, for justice and temperance. In other surgical situations, temperance and justice will be essential (for example, when a cancer surgeon discovers unexpected metastases they need to employ self-control and an understanding of what is due to the patient in deciding whether to continue with the procedure). Similarly, temperance and justice will be necessary when giving a patient information to enable them to decide whether to consent to the transplant.

Practical wisdom will also direct the researcher into thinking about the skills and virtues that they need to be successful, and how they will require them. This will include training or development, or the recruitment of others with the necessary attributes.

### Other virtues

Many other virtues have been described. One of the most pertinent for academic clinicians is clinical curiosity, the desire to ask questions about the patient, their condition, their situation and what the literature says.^58–60^ In the context of academic medicine, clinical curiosity provokes the individual to carry out research and to expand knowledge. Clinical curiosity has been described to be what makes an excellent clinician,^58^ and one of the important roles of academic clinicians may be to provoke clinical curiosity in other medical professionals.

Curiosity will be important in the design of the research project, understanding what others have done. It may also encourage engagement with people who have different viewpoints (medical, scientific, ethical and political), to understand if there is anything that can be learned from them. In a large research team it will work against ‘group think’, seeking to question things from different angles.

## What does virtue ethics offer?

Virtue ethics is complementary to the deontological and consequentialist frameworks for ethical reasoning. One of its features is that, unlike these approaches, it promotes the pursuit of excellence rather than ‘good enough’ actions or behaviour.^61^ It also requires the individual to take responsibility for their ethical conduct. It also provides a framework for the researcher to reflect on their character and how to develop it to be the best that they can be. However, it is less successful as a framework for regulation, it is difficult to discipline someone for having a ‘bad character’ (though many of the decisions that we make about whether to trust or work with someone will involve character judgements).

It is enlightening to consider how virtue ethics might be used to reflect on the introduction of cardiac allotransplantation. There was a rather undignified race to carry out the first heart transplant, which happened in South Africa in December 1967.^62^ The recipient initially did well but died after 18 days. The next few years there was rush of transplants, by the end of 1968 102 cardiac allotransplants had been performed.^29^ This has been described as ‘driven by a combination of intense publicity, surgical machismo, and national chauvinism’,^29^ with many of the centres lacking necessary expertise, for example in immunosuppression. Only a handful of recipients survived more than 6 months. In addition to the harm done to patients, this resulted in reputational damage to transplant research with a moratorium on cardiac transplants from November 1970.^29^

Looking at this through the lens of virtue ethics it would appear that some of the surgeons involved had lost sight of their *telos*. Their purpose seems to have been more about fame, reputation and national pride. The overall transplant community had lost sight of the health of their patient and the advance of science (I am not judging individuals; some of those involved were appropriately motivated by the needs of their patients and struggled with the ethics to do right thing). Many of those involved failed to orchestrate their virtues. In many cases it appears that they overplayed courage and did not apply sufficient temperance (possibly believing that their technical abilities would ensure success against all odds). The virtue of justice was underplayed; the need to demonstrate success meant that the needs of patients were not adequately considered. Overall, there was an excess of hubris. As we move towards cardiac xenotransplantation there is a need to remember the lessons of this unedifying period in medical and surgical history and consider more carefully what it means to be a ‘good’ researcher or clinician contributing to this work (understanding good as embracing both moral and practical outcomes).

## Conclusion

Xenotransplantation has long promised a revolution in the treatment of end stage organ failure. That promise has been a long time in coming! However, recent advances have shown that there is a real prospect of organs from pigs being effective in the treatment of human disease.

The ethical considerations in xenotransplantation have been long discussed. In general, the ethical debate has been couched in emotional, consequentialist or deontological terms. These are all important considerations. However, an alternative framework is the use of virtue ethics, which would consider the purpose and character of the clinicians, scientists and others involved in the process. This allows those involved in research to reflect on why they are doing the work, what character that they need to do it well and the wisdom to orchestrate their virtues and skills appropriately.

In this paper this has been discussed in the context of xenotransplantation. The same lens for virtue ethics also is relevant to all clinical academics who have to align their purpose of helping their patient to flourish and of developing wider understanding and benefit.
